# The feasibility of contrast enhanced ultrasonography (CEUS) in the diagnosis of non-cardiac thoracic disorders of dogs and cats

**DOI:** 10.1186/s12917-017-1061-0

**Published:** 2017-05-25

**Authors:** N. Linta, M. Baron Toaldo, G. Bettini, A. Cordella, M. Quinci, P. Pey, V. Galli, M. Cipone, A. Diana

**Affiliations:** 1 Department of Veterinary Medical Sciences, Alma Mater Studiorum – University of Bologna, Via Tolara di Sopra 50, I-40064 Ozzano Emilia, Bologna Italy; 2 ANTECH Imaging Services, 17672-B Cowan Avenue, Irvine, CA 92614 USA; 3 Freelance sonographer, Rome, Italy

**Keywords:** Small animals, Contrast medium, Ultrasonography, Tumor, Lung, Mediastinum

## Abstract

**Background:**

This study describes the feasibility of Contrast Enhanced Ultrasonography (CEUS) in the diagnostic work-up of non-cardiac thoracic disorders of small animals. The second aim is to assess the usefulness of CEUS as a direct guide for sample procedures.

**Results:**

Forty animals, 28 dogs and 12 cats, were included in the study. Thoracic disorders included 23 pulmonary lesions [primary carcinoma (14), lymphoma (1), sarcoma (1), histiocytic sarcoma (1), abscess (1) and pneumonia (5)] and 17 mediastinal lesions [lymphoma (8), thymoma (3), mesothelioma (1), melanoma (1), carcinomatous lymphadenopathy (1), mixsosarcoma (1), lipoma (1), and abscess (1)]. The majority of neoplastic pulmonary lesions showed an inhomogeneous distribution of contrast medium, whereas inflammatory lesions had a homogenous distribution with typical pulmonary vessels ramification. The majority of mediastinal malignant lesions showed an inhomogeneous distribution pattern. The lung and mediastinal abscesses had peripheral enhancement of the wall with an avascular center. All cytological and biopsy samples obtained after CEUS were diagnostic. Quantitative analysis, performed in 19/23 pulmonary lesions, showed a statistically significant difference (*P* < 0.0001) between the arrival time of the malignant (7.27 s - range 4.46–13.52 s) and benign (4.52 s - range 2.87–6.06 s) pulmonary lesions.

**Conclusions:**

CEUS may be a useful tool for the evaluation of non-cardiac thoracic lesions. The contrast medium allows for the precise definition of lesion edges, the presence of necrotic areas, and the distribution of pulmonary vessels.

Based on our preliminary results, the use of ultrasonographic contrast medium can be recommended for improving the diagnostic usefulness of cytology and biopsy sampling, because CEUS may help to define necrotic areas from viable tissue.

## Background

Thoracic ultrasounds are an important supplemental imaging modality in the diagnosis of non-cardiac thoracic disorders [1–3]. In veterinary medicine, several studies have addressed the usefulness of conventional ultrasounds for evaluating interstitial lung disease, during chronic conditions and emergency settings [4–6]. In the latter scenario, conventional ultrasounds have become of essential aid to increase diagnostic accuracy in traumatized patients or in animals with lung edema [7–9]. Since sound waves do not propagate through the air, this technique is limited to the evaluation of structures within or adjacent to the thoracic wall or to the presence of pleural effusion that creates an acoustic window allowing visualization of the intrathoracic structures [1–3]. Thoracic ultrasonography is also employed to guide interventional procedures (i.e., thoracentesis or biopsy of mediastinal and peripheral lung masses) to increase efficiency and safety [3].

In human medicine, the role of contrast-enhanced ultrasonography (CEUS) in the diagnosis of pathological conditions of the thorax and the appearances of different non-cardiac thoracic lesions have been reported [10–15]. Based on the dual arterial blood supply of the lung (i.e., pulmonary and bronchial arteries), the behavior of contrast medium in the lung parenchyma is similar to the liver (i.e., dual vascular supply characterized by arterial and portal feeding). This means that intravenously injected contrast medium reaches the lungs in the early phase (2–6 s after inoculation) via the pulmonary arteries and immediately after (7–20 s after inoculation) via the bronchial arteries and is eliminated in the late phase through the pulmonary and bronchial veins [11, 15].

The two most important parameters used in human medicine to discriminate benign from malignant lesions is the time until contrast enhancement and the distribution of the contrast medium. Specifically, benign lesions such as pneumonia commonly have a short time to enhancement and show intense and homogenous tissue enhancement with a regular, tree-like distribution of pulmonary arterial vessels. On the contrary, neoplastic lesions often present neoangiogenesis originating mainly from the bronchial arteries, creating an inhomogeneous tissue enhancement because of chaotic vascularization [11, 15].

In human medicine, CEUS is also used as a guide for transthoracic biopsy to allow for the differentiation of necrotic tissue from perfused tissue to identify the most appropriate sampling sites [16–18].

To our knowledge, information regarding the use of CEUS for the evaluation of non-cardiac thoracic disorders in veterinary literature is limited to a series of three cases of lung lobe torsion [19] and an abstract collecting of 15 cases with peripheral pulmonary and mediastinal mass lesions [20]. The first aim of this study was to prospectively assess the feasibility of CEUS in the characterization of non-cardiac thoracic disorders of small animals and to describe the CEUS pattern of pulmonary and mediastinal lesions. The second aim was to assess the usefulness of CEUS as a direct guide for sampling procedures.

## Methods

### Study population

Animals were prospectively recruited at the Veterinary Teaching Hospital of the University of Bologna from January 2010 to March 2015. Patients were enrolled into the study if they had a diagnosis of non-cardiac intra-thoracic lesions observed with conventional ultrasound, including peripheral lung lesions abutting the pleural surface and mediastinal lesions. Moreover, each patient must have had CEUS of the thoracic lesion performed and of sufficient quality to be subsequently evaluated. A final diagnosis of the lesion, through cytological or histological examinations, was a mandatory inclusion criterion.

Exclusion criteria were:

1) Technically inaccessible lesions (lesions that could not be visualized or poorly visualized with conventional ultrasound; or lesions not reachable for sample collection);

2) Severe dyspnea that could not be stabilized within 24 h from ultrasonographic exam;

3) CEUS not performed or of poor quality;

4) Inconclusive or missing final pathological diagnosis;

5) Clinical and radiographic features suggestive of cardiac disorders.

Informed owner consent was obtained before each CEUS examination and all procedures were performed in accordance with Italian laws on animal care.

### Ultrasound procedures and analysis

All procedures were conducted by the same experienced ultrasonographer (AD), using a real-time ultrasound machine^1^ equipped with a Micronvex probe (5–8 MHz), a broadband curved array transducer (2–5 MHz), and a linear array transducer (3–9 MHz). Conventional ultrasound and CEUS examination were performed within 24 h from admission and patient stabilization when needed. All patients received a survey of thoracic radiographs during their diagnostic work up. The time between radiographic and ultrasonographic study was less than 24 h. The animals were awake and restrained manually during the ultrasonographic procedures. Baseline grey-scale and color imaging was initially performed to localize the thoracic lesion and determine the optimal intercostal window for subsequent assessment. Each lesion was characterized by location, shape, size, echogenicity, and echotexture. The ultrasound setting was then switched to the Pulse Inversion Harmonic and Power Modulation combined (PMPI) with a low mechanical index set (0.07) for the contrast enhanced ultrasound. The gain setting was regulated to obtain anechoic thoracic lesions at baseline and a focal zone was placed just below the lesions to minimize microbubble destruction. The frame rate of CEUS loops varied between 10 and 30 Hz. With the transducer held in a fixed stable position, a bolus injection of SonoVue^2^ (0.5 ml for cats and 0.05 ml/kg for dogs) was manually injected through an indwelling cephalic venous catheter (20 or 22G), followed immediately by a rapid bolus of 4 mL saline. The images were recorded as cine-segments in DICOM format of 120 s starting from the time of contrast medium injection and then transferred to a personal computer.

Show Case software^3^ was used to review the images and to export selected frames for qualitative analysis. The distribution of the contrast medium enhancement within the lesions was evaluated subjectively as homogeneous or inhomogeneous during contrast uptake (wash-in phase), at peak intensity and during the progressive wash-out of contrast medium. Other specific findings were noted such as linear hyperechoic structures due to pulmonary vessel enhancement, anechoic areas inside the lesion, or peripheral enhancement of the lesion. Commercial software^4^ was used for quantitative computerized analysis of the contrast medium blood pool phase of the pulmonary lesions as previously described by our group for other organs [21, 22]. A region of interest (ROI) was manually drawn in the lesion as large as reasonably achievable, avoiding adjacent major vessels, and the presence of residual pulmonary air. The ROI was maintained in the same position by the motion compensation tool from QLAB software. This tool prevents the displacement of the ROI during respiratory motion. Furthermore, the ROI was adjusted manually on those frames severely affected by respiratory motion. Artefactual data from adjacent pulmonary tissue that moved into the ROI during respiratory motion were manually removed from the final dataset to reduce noise. The raw data obtained from each patient were plotted in quantitative time-intensity curves after the data were fitted to a mathematical model curve. The following perfusion variables were recorded: *arrival time* (AT, expressed in sec), defined as the time when the contrast signal is increasing to greater than about 10% of the peak intensity*; time to peak from injection* (TTPinj, expressed in sec); *time to peak from initial rise* (TTPinr, expressed in sec) calculated as TTPinj – AT; *peak intensity* (PI, expressed in arbitrary units [a.u.]) was defined as the highest intensity value minus baseline intensity before the initial rise; and *wash-in rate* (Wi, expressed in a.u./sec) was calculated with the data 10% above baseline intensity (BI) up to 85% of the peak value using a linear regression.

### Sample collection

**Fig. 1:**
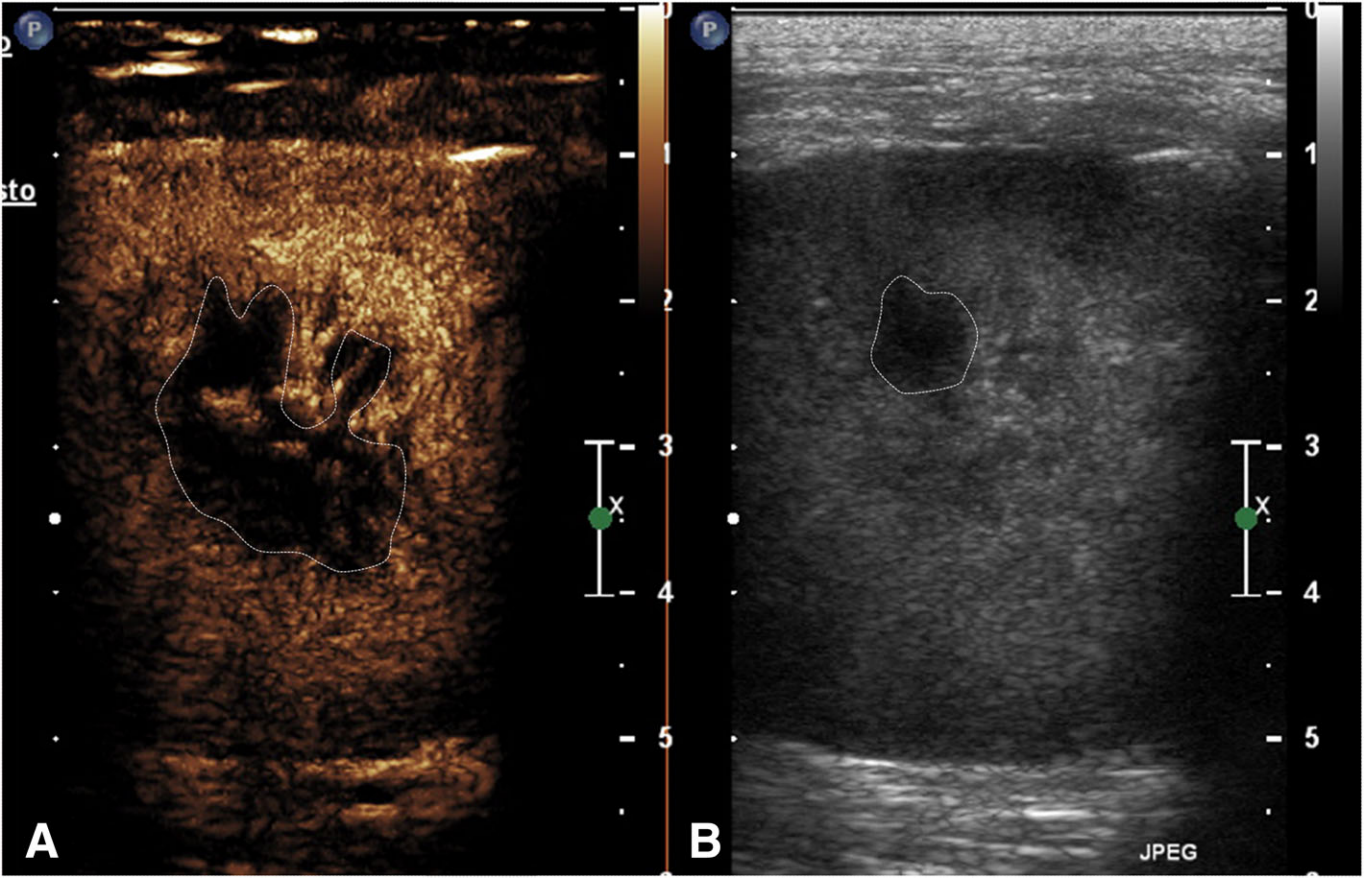
Transverse scan of a domestic shorthair cat with mediastinal thymoma in the contrast enhancement image (**a**) and the grey scale image (**b**) respectively. During the peak enhancement the contrast medium revealed a greater irregular avascular area (dotted line) (**a**) in comparison to grey scale image (**b**)

Immediately after the completion of CEUS, dogs were sedated and ultrasound-guided fine needle aspiration (FNA) and/or tissue core biopsy (TB) were performed. The information obtained from the contrast medium distribution was used to select sampling sites (areas showing contrast enhancement within the lesion; Fig. 1). Fine needle aspiration of the lesions was performed using a 22-gauge spinal needle,^5^ whereas TB was carried out with 14- (for dogs) and 16- (for cats) gauge Tru-Cut-type semiautomatic needles with a 20-mm specimen notch at the tip.^6^ In specific cases, cytological and/or histological sampling though bronchoscopy was allowed, providing that this was diagnostic. The endoscopic procedure was performed on the same day as CEUS.

### Statistical analysis

The data obtained from the quantitative perfusion analysis of the pulmonary lesions were regressed for significance of linearity using a D’Agostino-Pearson test and expressed as median and range. A Mann-Whitney test was used to compare the results of perfusion parameters between malignant and benign pulmonary lesions. A *P*-value less than 0.05 was considered statistically significant. Statistical analyses were performed using a commercial software package.^7^

## Results

Forty animals (28 dogs and 12 cats) with intra-thoracic diseases were included in the study. In the group of dogs, mixed breeds were overrepresented (13/28 dogs). There were three Labrador retrievers, two Rottweilers, two Basset hounds, and one each of the following breeds: Golden retriever, Great Dane, German shepherd, Dogue de Bordeaux, Weimaraner, Cocker spaniel, Bernese mountain dog, and English setter. There were 14 intact and two neutered males, and four intact and eight neutered females. The mean age was 8.6 ± 3.5 years. Among the group of cats, there were 11/12 domestic shorthair cats and one Chartreux, made up of two intact and four neutered males, and two intact and four neutered females. The median age was 8.8 ± 3.8 years.

A total of 40 lesions were examined, including 23 pulmonary lesions and 17 mediastinal lesions. The diameter of the pulmonary lesions ranged from 1 to 18.3 cm. Four lesions were located in the left apical lobe, two in the right apical lobe, five in the right medial lobe, seven in the left diaphragmatic lobe, and 10 in the right diaphragmatic lobe. The diameter of the mediastinal lesions ranged from 1.6–12.4 cm. Fifteen lesions were located in the cranial mediastinum and two in the caudal mediastinum.

Of the 40 cases, 32 had malignant lesions and eight had benign lesions. For the malignant lesions, the diagnosis was obtained by cytological and histological confirmation following CEUS on 25 and seven cases, respectively. In cases of benign lesions, the diagnosis was based on cytology following CEUS in three patients and on histology following bronchoscopic biopsy in the other five cases. All cytological and biopsy samples obtained after CEUS were diagnostic. Table 1 reports the final diagnoses.

**Table 1:** Final diagnosis in the malignant and benign patient groups

Localization	Final Diagnosis	Number of lesions	Number of dogs	Number of cats
LUNG	Malignant			
	Carcinoma	14	10	4
	Sarcoma	1	1	0
	Histiocytic sarcoma	1	1	0
	Lymphoma	1	0	1
	Benign			
	Pneumonia	5	5	0
	Abscess	1	1	0
MEDIASTINUM	Malignant			
	Lymphoma	8	4	4
	Thymoma	3	2	1
	Myxosarcoma	1	1	0
	Mesothelioma	1	1	0
	Carcinomatous lymphadenopathy	1	1	0
	Melanoma	1	1	0
	Benign			
	Lipoma	1	0	1
	Abscess	1	0	1
Total		40	28	12

Tables 2 and 3 summarize the results of baseline grey-scale ultrasonography of pulmonary and mediastinal lesions, respectively. The malignant pulmonary lesions had a round-oval shape and hypoechoic appearance. The echotexture was inhomogeneous in all cases. Five benign pulmonary lesions showed a triangular shape with a liver-like appearance associated with images of air and fluid bronchograms. Most of the malignant mediastinal lesions were masses with a diameter > 50 mm, a hypoechoic appearance and an inhomogeneous echotexture.

**Table 2:** Baseline grey-scale ultrasonography of 23 pulmonary lesions

Pulmonary lesions		Location	Shape	Margins	Echogenicity	Echotexture	Size
Malignant	Carcinoma (14)	LAL (3) LAR (1) LDL (4) LDR (4) MULTIFOCAL (2)	Rounded (5) Oval (9)	Well-defined (14)	Hypoechoic (14)	Inhomogeneous (14)	< 50 mm (6) > 50 mm (8)
	Sarcoma (1)	LDL	Rounded	Well-defined	Hypoechoic	Inhomogeneous	> 50 mm
	Histiocytic sarcoma (1)	LDL	Oval	Well-defined	Heterogeneously anechoic	Inhomogeneous	< 50 mm
	Lymphoma (1)	MULTIFOCAL	Rounded	Well-defined	Hypoechoic	Inhomogeneous	< 50 mm
Benign	Pneumonia (5)	LAL (1) LMR (1) LDR (1) MULTIFOCAL (2)	Triangular	Well-defined (3) Ill-defined (2)	Hypoechoic (5)	Homogeneous (4) Inhomogeneous (1)	> 50 mm (1) < 50 mm (4)
	Abscess (1)	LDR	Oval	Capsulated	Heterogeneously anechoic	NA	< 50 mm

*LAL* apical left lobe, *LDL* diaphragmatic left lobe, *LAR* apical right lobe, *LMR* medium right lobe, *LDR* diaphragmatic right lobe

**Table 3:** Baseline grey-scale ultrasonography of 17 mediastinal lesions

Mediastinal lesions		Location	Shape	Margins	Echogenicity	Echotexture	Size
Malignant	Lymphoma (8)	Cr Med (8)	Rounded (3) Oval (5)	Well-defined (8)	Hypoechoic (8)	Inhomogeneous (7) Homogeneous (1)	< 50 mm (3) > 50 mm (5)
	Thymoma (3)	Cr Med (3)	Oval (3)	Well-defined (3)	Hypoechoic (3)	Inhomogeneous (3)	> 50 mm
	Myxosarcoma (1)	Cr Med	Oval	Well-defined	Hypoechoic	Inhomogeneous	> 50 mm
	Mesothelioma (1)	Cr Med	Oval	Well-defined	Hypoechoic	Inhomogeneous	< 50 mm
	Carcinomatous lymphadenopathy (1)	Cr Med	Oval	Well-defined	Hypoechoic	Inhomogeneous	< 50 mm
	Melanoma (1)	Cr Med	Oval	Well-defined	Hypoechoic	Inhomogeneous	> 50 mm
Benign	Abscess (1)	Cau Med	Oval	Well-defined	Heterogeneously anechoic	NA	< 50 mm
	Lipoma (1)	Cau Med	Oval	Well-defined	Hypoechoic	Homogeneous	< 50 mm

*Cr Med* Cranial Mediastinum, *Cau Med* Caudal Mediastinum

The time for the entire contrast enhanced procedure (from setting the machine, and preparing and injecting the contrast until the end of the cine-segment) was five min. No adverse effects were noticed in any of the dogs during or after injection of the contrast medium. Qualitative evaluation was possible in all subjects imaged.

**Fig. 2:**
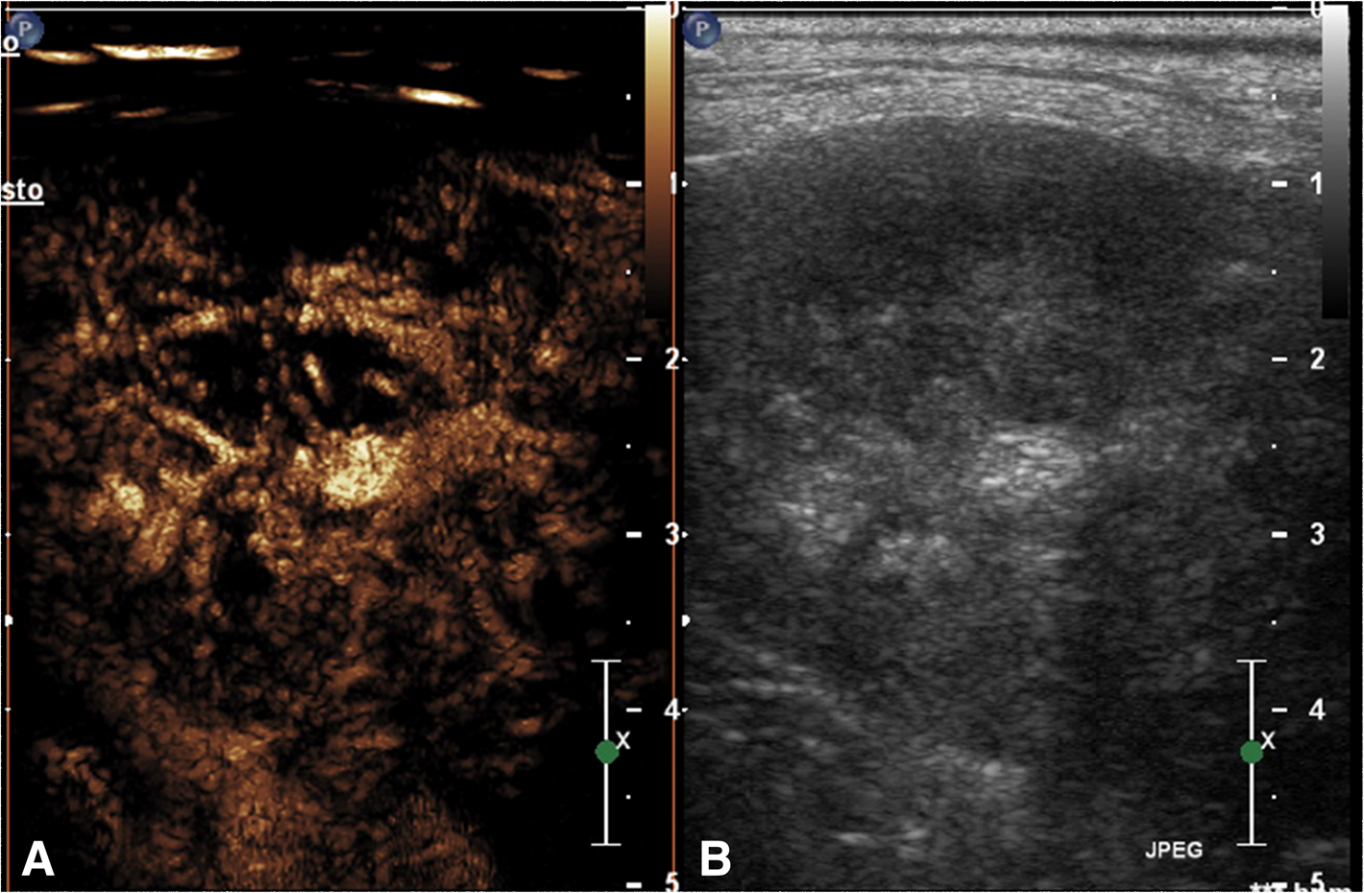
Transverse scan of a cocker spaniel dog with pulmonary carcinoma in the contrast enhancement image (**a**) and the grey scale image (**b**), respectively. At peak intensity the mass showed an irregular distribution of the vessels associated with an inhomogeneous enhancement

**Fig. 3:**
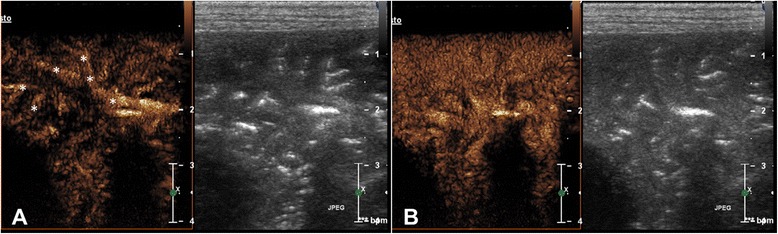
Longitudinal scan of right caudal pulmonary lobe of a crossbreed dog with pneumonia at wash in (**a**) and at peak intensity (**b**). Each image illustrates contrast enhancement on the left and the grey scale image on the right. (**a**) There is an intense enhancement of the pulmonary arteries (asterisks) with a typical tree-like ramification. (**b**) Intense and homogeneous enhancement of the entire lobe at peak intensity

**Fig. 4:**
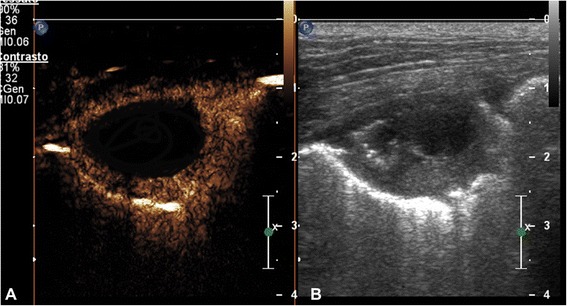
Transverse scan of a right caudal pulmonary lobe of an English setter dog with pulmonary abscess in the contrast enhancement image (**a**) and the grey scale image (**b**), respectively. At peak intensity the lesion was characterized by a peripheral enhancement with avascular center

Concerning pulmonary diseases, the majority of malignant lesions (16/17) showed an inhomogeneous enhancement at PI with anechoic areas inside the lesions (Fig. 2). Four benign lesions (4/6) showed a homogenous enhancement with linear hyperechoic pulmonary structures branching out from the periphery (Fig. 3a and b). One benign pulmonary lesion showed a rim enhancement of the lesion with an anechoic centre (Fig. 4). Table 4 summarizes all CEUS patterns of pulmonary lesions.

**Table 4:** CEUS findings of 23 pulmonary lesions

Pulmonary lesions		Wash-in	Peak intensity	Wash-out
Malignant	Carcinoma (14)	Homogeneous (1) Inhomogeneous (13)	Homogeneous (1) Inhomogeneous (13)	Inhomogeneous (14)
	Sarcoma (1)	Inhomogeneous	Inhomogeneous	Inhomogeneous
	Histiocytic sarcoma (1)	Inhomogeneous	Inhomogeneous	Inhomogeneous
	Lymphoma (1)	Inhomogeneous	Inhomogeneous	Inhomogeneous
Benign	Abscess (1)	Homogeneous enhancement of wall and avascular center	Homogeneous enhancement of wall and avascular center	Inhomogeneous enhancement of wall and avascular center
	Pneumonia (5)	Homogeneous with normal vessels ramification (4)	Homogeneous (4)	Inhomogeneous with normal vessels ramification (5)
		Inhomogeneous with normal vessels ramification (1)	Inhomogeneous (1)	

**Fig. 5:**
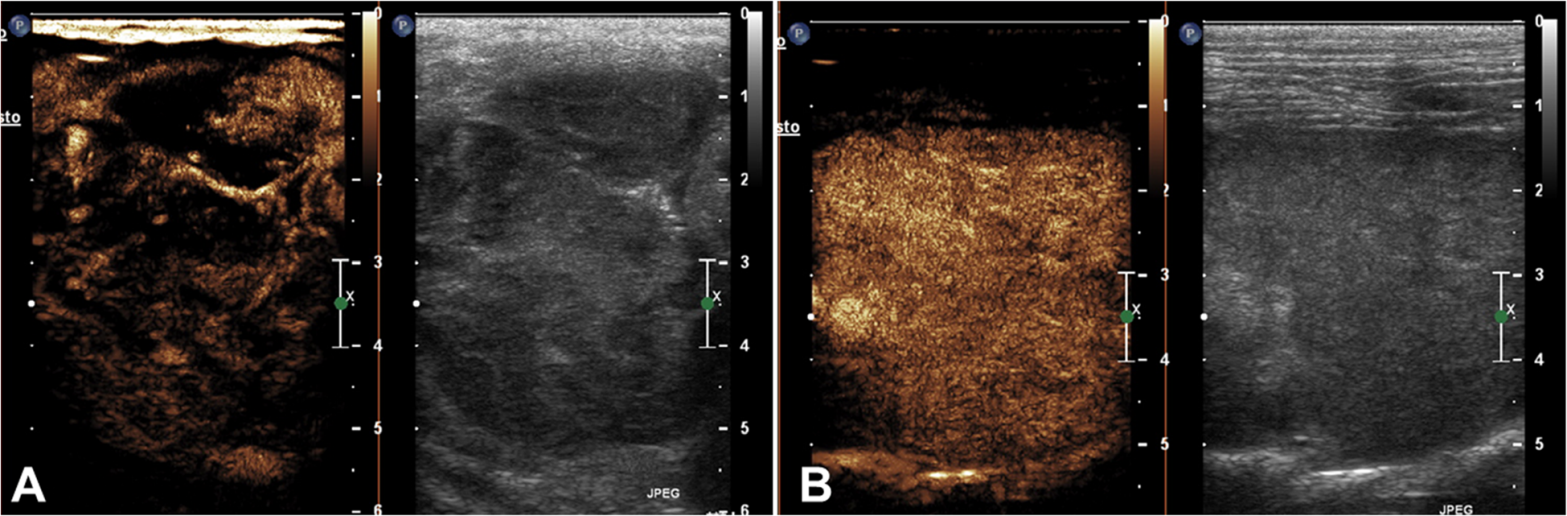
Transverse scan of a thymoma in a crossbreed dog (**a**) and of a mediastinal lymphoma in a domestic short hair cat (**b**). Each image illustrates contrast enhancement on the left and the grey scale image on the right. (**a**) There is an inhomogeneous enhancement of the mass at peak intensity with avascular areas. (**b**) The mass showed an intense and homogenous enhancement at peak intensity

Most of the malignant mediastinal lesions (10/15) showed an inhomogeneous distribution of the contrast medium at PI characterized by extended areas lacking contrast medium (Fig. 5a), whereas the other malignant lesions (5/15) had a homogenous perfusion pattern (Fig. 5b). Inhomogeneous enhancement and peripheral enhancement of the lesion with an anechoic center was found in the other two benign lesions, respectively (Table 5).

**Table 5:** CEUS findings of 17 mediastinal lesions

Mediastinal lesions		Wash-in	Peak intensity	Wash-out
Malignant	Lymphoma (8)	Homogeneous (4)	Homogeneous (4)	Inhomogeneous (8)
		Inhomogeneous (4)	Inhomogeneous (4)	
	Thymoma (3)	Homogeneous (1)	Homogeneous (1)	Inhomogeneous (3)
		Inhomogeneous (2)	Inhomogeneous (2)	
	Mixsosarcoma (1)	Inhomogeneous	Inhomogeneous	Inhomogeneous
	Mesothelioma (1)	Inhomogeneous	Inhomogeneous	Inhomogeneous
	Carcinomatous lymphadenopathy (1)	Inhomogeneous	Inhomogeneous	Inhomogeneous
	Melanoma (1)	Inhomogeneous	Inhomogeneous	Inhomogeneous
Benign	Abscess (1)	Homogeneous enhancement of wall and avascular center	Homogeneous enhancement of wall and avascular center	Inhomogeneous enhancement of wall and avascular center
	Lipoma (1)	Inhomogeneous	Inhomogeneous	Inhomogeneous

**Fig. 6:**
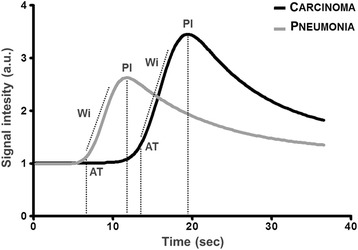
Signal intensity in arbitrary units (a.u.) as a function of time in seconds of two representative cases included in the study. Grey line refers to a crossbreed dog with pneumonia while the black line refers to a crossbreed dog with pulmonary carcinoma. Note the delayed contrast agent arrival (expressed as arrival time [AT]) shown by the carcinoma in comparison to the pneumonia. PI, peak intensity (expressed in arbitrary units [a.u.]); Wi, wash-in slope (expressed in a.u./sec)

Quantitative analysis was performed in 19/23 pulmonary lesions (Table 6). Excessive respiratory motion precluded quantitative analysis in the remaining four pulmonary lesions, all of which were malignant. There was a statistically significant difference between the AT of the malignant and benign pulmonary lesions (*P* < 0.001) (Fig. 6).

**Table 6:** Quantitative computerized analysis of the contrast medium blood pool phase of 19 pulmonary lesions

Pulmonary lesions	Malignant median (range)	Benign median (range)	*P*-value
Number of lesions	13	6	
AT	7.27 (4.46–13.52)	4.52 (2.87–6.06)	< 0.001*
TTPinj	13.78 (10.24–24.50)	9.83 (4.70–13.35)	0.059
TTPinr	5.91 (3.79–14.87)	4.55 (1.83–9.56)	0.236
PI	2.37 (0.68–11.87)	3.17 (1.63–8.56)	0.273
Wi	0.25 (0.10–1.99)	0.45 (0.20–1.96)	0.242

*AT* Arrival Time, *TTPinj* time to peak from injection, *TTPinr* time to peak from initial rise, *PI* peak intensity, *Wi* wash-in rate

*Statistically significant

## Discussion

This is the first study evaluating the use of CEUS in the characterization of non-cardiac thoracic disorders in small animals. Contrast-enhanced ultrasound appeared to be feasible when examining pulmonary and mediastinal lesions in dogs and cats, giving useful information for the clinician. No adverse reactions have been reported and even patients with respiratory distress, once stabilized, could be submitted to CEUS examination safely. Different patterns of enhancement have been observed for various disease processes, and the timing of contrast uptake varied between benign and malignant pulmonary lesions. Moreover, the contrast distribution within the lesion could help in identifying areas of poor or null perfusion, likely referable to regions of ischemia or necrosis; therefore, guiding sample collections for cytological or histological examination and possibly increasing diagnostic accuracy.

While in veterinary medicine little has been published concerning CEUS for the evaluation of thoracic lesion [19, 20], several studies are available in the human literature [11–15]. The CEUS patterns of our inflammatory pulmonary lesions were similar to those previously reported in humans [10, 11, 13, 14]. In particular, in pneumonia, the CEUS pattern was characterized by a rapid distribution of the contrast medium into the branches of pulmonary arteries giving a typical treelike appearance. Subsequently, a homogeneous and intense tissue enhancement during the parenchymal phase was seen. Conversely, the majority of our neoplastic pulmonary lesions showed an inhomogeneous distribution of the contrast media with different sized avascular areas because of chaotic neoplastic neoangiogenesis and necrosis.

In human medicine, lung cancer is usually characterized by sparse tissue enhancement suggesting a bronchial arterial supply [10, 11]. In particular, tumor neoangiogenesis in human lung cancer is reported to arise from bronchial arteries while pulmonary arteries seem to have no or a low capacity for neoangiogenesis [10]. However, some lung cancer subentities, such as bronchioloalveolar carcinoma and adenocarcinoma, may instead present with a pneumonia-like contrast-enhanced sonographic patterns because of a pulmonary arterial supply [10–12, 14, 15]. We did not find different CEUS behavior in our neoplastic pulmonary group, and all lung cancer cases showed an inhomogeneous distribution of the contrast medium. The different CEUS behavior of adenocarcinoma between dogs and humans may be explained by the proliferation of bronchial arteries in canine primary and metastatic pulmonary tumors while the pulmonary arteries are displaced or distorted by tumor mass or occluded by thrombi [23]. Moreover, the low number of cases enrolled in the present study might also contribute to the discrepancies observed between dogs and human patients.

Concerning mediastinal lymphoma, we found a different CEUS pattern related to the dimension of the lesions. Lesions with a diameter less than 50 mm showed a homogeneous enhancement, while those greater than 50 mm were characterized by an inhomogeneous pattern with avascular areas. This CEUS behavior is similar to that reported in human medicine where lymphomas have a more variable CEUS appearance [24, 25]. Our other mediastinal tumors (thymoma, myxosarcoma, melanoma, carcinomatous lymphadenopathy, and mesothelioma) showed an inhomogeneous enhancement and all of these tumors had a diameter greater than 50 mm.

In our cases, qualitative assessment of contrast medium distribution was useful for differentiating benign from malignant lung lesions. All pneumonia cases showed a homogeneous enhancement with the pulmonary arteries observed as linear hyperechoic structures with greater enhancement compared to the parenchyma, whereas the majority of lung neoplasia (16/17 cases) had an inhomogeneous pattern with avascular areas. These findings suggest that CEUS can help in differentiating between lesions of different nature. If CEUS could increase the diagnostic accuracy of conventional ultrasound is unable to be assessed based on the present study. The shape of the lesions, the distribution and ramification of bronchi and vessels, and the echo-texture observed with conventional ultrasound were already highly suggestive of an inflammatory or neoplastic process, as already described in veterinary literature [3]. In the present study, we did not include patients with pulmonary infiltrative tumors that might mimic pneumonia (i.e. round cell neoplasia). It would be an intriguing aspect of CEUS, if this technique could help in differentiating these subtle processes that cannot otherwise be easily discriminated using conventional ultrasound. A unique type of lesion is represented by the abscess. This process organizes as a mass that could be misdiagnosed with a solid tumor when using conventional ultrasound. In this specific case, CEUS appears particularly helpful, since it shows a characteristic pattern of contrast distribution with intense peripheral rim enhancement and anechoic center that is not usually present in solid tumors, unless extensive intralesional necrosis occurs sparing exclusively the peripheral rim (this condition is unlikely to occur with this tidy fashion). In our case report all abscesses had a unique CEUS pattern, similar to what was observed in human lungs and livers [18, 26].

In human medicine, the differentiation between benign and malignant pulmonary lesions is based on the different hemodynamic behaviors of the contrast medium by measuring the time to enhancement. Benign lesions have a shorter time to enhancement (due to pulmonary artery supply: 2–6 s) compared to malignant lesions (due to bronchial artery supply: 7–20 s) [10, 11]. This result has been recently reported in a single abstract describing the CEUS ultrasonography in fifteen small animals affected by peripheral pulmonary and mediastinal mass lesions [20]. Particularly, the authors described a significant difference in time to enhancement between carcinomas and sarcomas and between carcinomas and inflammatory lesions [20]. Quantitative analysis of our pulmonary lesions showed a statistical difference in the arrival time of the contrast medium between inflammatory and neoplastic groups. This finding is in agreement with that reported in human medicine and can be explained by the difference in vascularization between the two groups of lesions. Vascularization in benign inflammatory lesions is mainly related to pulmonary arteries, whereas in neoplastic lesions it is a consequence of bronchial origin neovascularization.

All the FNA or TB performed after administration of the contrast medium were successfully diagnostic. In human literature, several studies demonstrated the utility of CEUS as a guide for transthoracic biopsy to avoid necrotic areas inside large masses. Its diagnostic accuracy was reported around 93.6% [16, 27]. Although real time ultrasonography can disclose the presence of necrosis inside the lesion and sonographic guidance is considered particularly useful in the diagnosis of pulmonary masses with necrotic centers, inadequate specimens have been reported in 9–26% of cases when the necrotic component is large [28]. In our cases, avascular areas consistent with necrosis were more frequently found in masses with a diameter greater than 50 mm. This value is also reported in human medicine causing false negative results during sample collections for cytological examination [16, 27].

This study has some limitations that need to be emphasized. First, the relatively low number of cases and type of diseases observed. Second, out of the 19 pulmonary lesions quantified, 13 were malignant and 6 were benign; this unbalance could have influenced the statistical analysis. Third, most of our final diagnoses were obtained by FNA, which limited the possibility of further classifying the lesions by histological type, especially for lung tumors. In human medicine, a different CEUS behavior of bronchioloalveolar carcinoma and adenocarcinoma in comparison to other lung tumors have been reported [10, 11, 14, 15]. Finally, although CEUS appears useful in characterizing intra-thoracic lesions of different natures, conventional ultrasound was already able to provide most of the information needed to hypothesize the benign or malignant behavior of the lesions. A larger number of cases and the characterization of different pathological processes are needed to understand if CEUS could outperform conventional ultrasound in discriminating between thoracic lesions.

## Conclusions

In conclusion, we have demonstrated the feasibility of CEUS in the evaluation of canine and feline lung and mediastinal disorders. The injection of a microbubble contrast medium allows for precise definition of lesion edges, the presence of necrotic areas, and the distribution of pulmonary vessels. Most of the pulmonary and mediastinal malignant lesions were characterized by an inhomogeneous distribution of the contrast medium, whereas homogenous tissue enhancement with regular branching distribution of pulmonary arterial vessels was seen with pneumonia. A distinctive contrast perfusion pattern for the different types of malignant lesions was not obtained and an increased number of cases will be necessary for characterization.

Based on our preliminary results, the use of CEUS can be recommended as a direct guide for sampling procedures to improve the diagnostic success of cytological and histological sampling.

## Abbreviations

AT: Arrival time
BI: Baseline intensity
CEUS: Contrast-enhanced ultrasound
FNA: Fine needle aspiration
PI: Peak intensity
ROI: Region of interest
TB: Tissue core biopsy
TTP inj: Time to peak from injection
TTP inr: Time to peak from initial rise
Wi: Wash-in rate

## Footnotes

1 iU22 ultrasound system, Philips Healthcare, Monza, Italy.

2 SonoVue, Bracco® diagnostic, Milano, Italy.

3 ShowCase software, Trillium Technology, Ann Arbor, MI.

4 QLAB quantification software, Philips Healthcare, Monza, Italy.

5 BD spinal needle, Becton Dickinson SA, Madrid, Spain

6 Tru-Cut, Hospital Service SPA, Latina, Italy

7 Prism 5®, GraphPad Software Inc., San Diego, CA.
